# A geospatial dataset of inland valleys in four zones in Benin, Sierra Leone and Mali

**DOI:** 10.1016/j.dib.2019.103699

**Published:** 2019-01-22

**Authors:** Justin F. Djagba, Amadou M. Kouyaté, Idriss Baggie, Sander J. Zwart

**Affiliations:** a Africa Rice Center (AfricaRice), Cotonou, Benin; b Centre Régional de la Recherche Agricole Sikasso, Institut d’Économie Rurale (IER), Sikasso, Mali; c Rokupr Agricultural Research Centre (RARC), Sierra Leone Agricultural Research Institute (SLARI), Rokupr, Sierra Leone; d International Water Management Institute (IWMI), Accra, Ghana

## Abstract

The dataset described in this data article represents four agricultural zones in West-Africa that are located in three countries: Benin, Mali and Sierra Leone. The dataset was created through a research collaboration between the Africa Rice Center (AfricaRice), Sierra Leone Agricultural Research Institute (SLARI) and the Institute for Rural Economy (IER). The dataset was compiled to investigate the potential for rice production in inland valleys of the three countries. The results of the investigation were published in Dossou-Yovo et al. (2017) and Djagba et al. (2018). The dataset describes the biophysical and socioeconomic conditions of 499 inland valleys in the four agricultural zones. In each inland valley data were collected through a focus group interview with a minimum of three farmers. In 499 interviews a total of 7496 farmers participated. The location of each inland valley was determined with handheld GPS devices. The geographic locations were used to extract additional parameters from digital maps on soils, elevation, population density, rainfall, flow accumulation, and distances to roads, market places, rice mills, chemical input stores, and settlements. The dataset contains 65 parameters in four themes (location, biophysical characteristics, socioeconomic characteristics, and inland valley land development and use). The GPS coordinates indicate the location of an inland valley, but they do not lead to the location of individual fields of farmers that were interviewed. The dataset is publicly shared as Supplementary data to this data article.

**Specifications table**

**Table:** 

Subject area	Agriculture, Geography, Sociology, Economics
More specific subject area	Africa, rural development, food security
Type of data	Table (Excel format)
How data were acquired	Face-to-face farmer groups interviews using a structured questionnaire; geographic locations obtained with handheld GPS devices; ancillary data extracted from maps using geographic coordinates
Data format	Raw,
Experimental factors	Data set was cleaned from duplications, data entry errors, incomplete responses, and wrongly GPS recorded coordinates
Experimental features	Inland valleys in four zones were randomly selected. A group of minimally 3 farmers from the selected inland valley was interviewed.
Data source location	Data are available for four regions (see also Fig. 1):
	1. Mono and Couffo departments (Benin) 2. Ouémé River upper catchment (Benin) 3. Sikasso and Kadiolo circles (Mali) 4. Bo and Kenema districts (Sierra Leone) The latitude and longitude coordinates of each inland valley are included in the dataset.
Data accessibility	Dataset is available with this article as Supplementary data
Related research article	Djagba, JF, LO Sintondji, AM Kouyaté, I Baggie, G Agbahungba, SJ Zwart, 2018. Predictors determining the potential of inland valleys for rice production in West-Africa. Applied Geography 96, pp. 86–97.

**Value of the data**

• A large multidisciplinary dataset comprising 499 inland valleys in three countries in West-Africa that cover location, biophysical characteristics, socioeconomic characteristics and inland valley exploitation.
• The dataset can be deployed to analyze the potential for agricultural development, to characterize diverse inland valley landscapes, to perform environment impact assessments, to classify land use from satellite imagery, etc.
• The dataset contributes to food security research and assessments in West-Africa and leads to further understanding of the diversity of agricultural systems and their potential to contribute to food production and income generation for the rural population.
• The dataset was deployed to assess the diversity and importance of inland valley agricultural systems to a regional scale in Sierra Leone [1].
• To expand regional coverage the data can be linked to similar surveys conducted in inland valleys in Niger state (Nigeria), entire Burkina Faso and southern Mali [3], [4].

## 1 Data

**Fig. 1:**
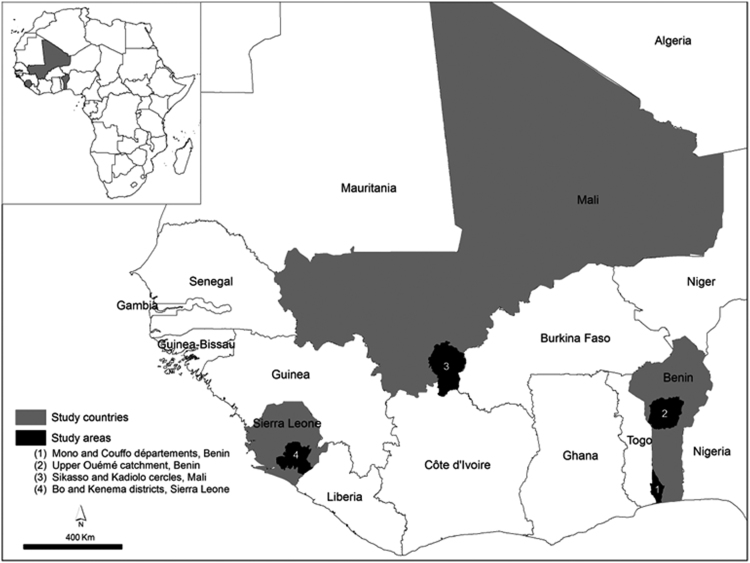
location of the four zones in West-Africa.

The dataset contains biophysical and socioeconomic information on 499 inland valleys in four zones in Benin, Mali and Sierra Leone (see Fig. 1). The inland valleys are geolocated with latitude/longitude coordinates. The parameters (Table 2), grouped in four themes (Table 1), were obtained from farmers’ responses during focus group interviews conducted in each of the 499 inland valleys between 2013 and 2014. Additional parameters were extracted from digital maps using the location of the inland valleys. Table 2 outlines the variables collected, and their source whether from the interviews or secondary spatial data sources.

**Table 1:** Themes, subject and total number of parameters in the inland valley dataset.

**Theme**	**Subjects**	**# of parameters**
1. Location	Coordinates (Lat/Lon)	1
2. Biophysical characteristics	Shape, width, soil type, surface water, groundwater, drainage	24
3. Socioeconomic characteristics	Farmers, ethnicity, farmer organization, markets, accessibility, land tenure	21
4. IV development and use	IV area, agriculture area, varieties, inputs, water supply, infrastructure	19

**Table 2:** Summary of the parameters included in the inland valley (IV) dataset.

**Subjects**	**Variable**	**Description**	**Unit**	**Type**	**Source**
Hydrological data	Floodurf	Flooding duration in inland valley (IV) fringe	Week	Quantitative	Field survey
	Floodurb	Flooding duration in IV bottom	Week	Quantitative	Field survey
	Flowacc	Flow accumulation (maximum)	Index	Quantitative	DEM/STRM^a^ (30 m)
	Watersou	Water flow source		Qualitative	Field survey
	Waterdur	Water flow duration		Qualitative	Field survey
	Watflodur	Water flow duration if temporary	Month	Quantitative	Field survey
	wtablemb	Emerging water table IV bottom duration	Month	Quantitative	Field survey
	wtablemf	Emerging water table IV fringe duration	Month	Quantitative	Field survey
	Wtablshb	Shallow water table IV bottom duration	Month	Quantitative	Field survey
	Wtablshf	Shallow water table IV fringe duration	Month	Quantitative	Field survey
	Drainage	IV drainage		Qualitative	Field survey
					
Topographical and climatic data	Shape	Transversal entrenchment shape		Qualitative	Field survey
	Elevation	Elevation (mean)	Meter	Quantitative	DEM /STRM (30m)
	Widthest	Estimated average width	Meter	Quantitative	Field survey
	Rainfall	Annual average rainfall	Millimeter	Quantitative	ARC2 for FEWS^b^
					
Soil data	OC	Soil organic carbon content	g kg^−1^	Quantitative	AfSoilGrids250m^c^
	Ntot	Total nitrogen	g kg^−1^	Quantitative	AfSoilGrids250m
	Exchbas	Exchangeable bases	Cmolc kg^−^^1^	Quantitative	AfSoilGrids250m
	Sand	Sand fraction at 30 cm depth	Percent	Quantitative	AfSoilGrids250m
	Clay	Clay fraction at 30 cm depth	Percent	Quantitative	AfSoilGrids250m
	pH	Soil pH in H_2_O	Index	Quantitative	AfSoilGrids250m
	Soilbot	Soil IV bottom		Qualitative	Field survey
	Soilfring	Soil IV fringe		Qualitative	Field survey
	Soilupslop	Soil upper slope		Qualitative	Field survey
					
Socio-economic and accessibility environment	Pavedrd	Nearest distance from IV to paved road	Meter	Quantitative	OSM^d^ & GoogleEarth
	Othroad	Nearest distance from IV to other road	Meter	Quantitative	OSM & GoogleEarth
	DistRd	Distance from IV to road	km	Quantitative	Field survey
	Settlement	Nearest distance from IV to a settlement	Meter	Quantitative	OSM & GoogleEarth
	Market	Nearest distance from IV to a market place	Meter	Quantitative	GPS location
	Ricemill	Nearest distance from IV to a rice mill	Meter	Quantitative	GPS location
	Store	Nearest distance from IV to a store of inputs	Meter	Quantitative	GPS location
	IVmarket	Road type between IV and market		Qualitative	Field survey
	Vilgmarket	Road type between village and market		Qualitative	Field survey
	IVmarketdis	Distance between IV and Market	km	Quantitative	Field survey
	Vilgmarketdis	Distance between village and market distance	km	Quantitative	Field survey
	Popden	Population density	Person.km^-2^	Quantitative	GPWV4^e^
	Landowner	Land ownership		Qualitative	Field survey
	Men	Number of male farmers in the IV	Person	Quantitative	Field survey
	Women	Number of female farmers in the IV	Person	Quantitative	Field survey
	Ethnig	Major ethnic groups		Qualitative	Field survey
	Migranpred	Predominance of the migrants in the use of IV		Qualitative	Field survey
	Landaccess	Access to land		Qualitative	Field survey
	Access	Accessibility of the IV		Qualitative	Field survey
	Seeds	Source of seeds		Qualitative	Field survey
	Otherinput	Source of other inputs		Qualitative	Field survey
					
Farm management practices data	Othcrop	Other crops cultivated in IV		Qualitative	Field survey
	Vegetable	Vegetable cultivation in IV		Qualitative	Field survey
	IVarea	Total area of the IV	Hectare	Quantitative	GPS data/GoogleEarth
	Exploitation	Mode of exploitation		Qualitative	Field survey
	Objective	Production objective		Qualitative	Field survey
	Agrisupport	Presence of agricultural support structure		Qualitative	Field survey
	Ivorganizat	Existence of IV farmers’ organization		Qualitative	Field survey
	Organizatyp	If yes, type of organization and if no, none		Qualitative	Field survey
	Dvlopd	IV development status		Qualitative	Field survey
	Soilmngt	Soil fertility management		Qualitative	Field survey
	Watersuply	Water supply		Qualitative	Field survey
	Irrigation	Irrigation water resource		Qualitative	Field survey
	Fields	Field development		Qualitative	Field survey
	Drainagpr	Drainage practices		Qualitative	Field survey
	Irrigationpr	Irrigation practices		Qualitative	Field survey
	Rsvegarea	Wet season vegetable cultivation area	Hectare	Quantitative	Field survey
	Dsvegarea	Dry season vegetable cultivation area	Hectare	Quantitative	Field survey
	Rsocroarea	Wet season other crops cultivation area	Hectare	Quantitative	Field survey
	Dsocroarea	Dry season other crops cultivation area	Hectare	Quantitative	Field survey

a Digital Elevation Model/Worldwide High-resolution Shuttle Radar Topography Mission (SRTM 30 m), URL: http://srtm.csi.org Data derivation were done in ArcGIS.

b African Rainfall Climatology Version 2 for Famine Early Warning Systems available at ftp.cpc.ncep.noaa.gov/fews/fewsdata/africa/arc2.

c Soil properties of African at 250 m, Soil Grids available at www.isric.org/data/AfSoilGrids250m.

d Open Street Map or digitizing from Google Earth. Layers derivation were done in ArcGIS.

e Gridded Population of the World (GPW) Version 4 in 2015, Center for International Earth Science Information Network (CIESIN).

The dataset is provided in Microsoft Excel format and contains seven sheets. The first sheet (*source*) provides citation information and refers to this data article. The second sheet (*variable explanation*) outlines the variables. After that the sheet location provides the unique identifier of each surveyed inland valleys and the geographic coordinates expressed in longitude/latitude. The unique identifier can be linked to the variables stored in four sheets, one for each of the four zones, called *Mali*, *Sierra Leone*, *Benin_Ouémé supérieur* and *Benin_Mono-Couffo* (Fig. 1).

## 2 Experimental design, materials and methods

This section provides a summary of the steps taken to develop the geospatial dataset. [2] provides a full description of the methodology that was followed.

Data collection was implemented in two phases. In the first phase, 499 inland valleys were identified in four zones in Mali, Benin and Sierra Leone. These were 100, 149, 100 and 150 inland valleys in Mono and Couffo departments (Benin), Upper Ouémé catchment (Benin), Sikasso and Kadiolo districts (Mali) and Bo and Kenema districts (Sierra Leone), respectively. These sites were visited by teams of trained surveyors equipped with a questionnaire and a GPS. Focus group interviews with at least three farmers operating in the inland valley were held and their responses were recorded. Focus groups existed of maximum 7496 farmers and on average 15 farmers participated in the focus group interviews. With the use of handheld GPS devices, the coordinates of the inland valleys were obtained.

In a second phase, the locations of the inland valleys were imported into a Geographic Information System and their quality was checked. Spatial information available in the public domain were downloaded and imported in a GIS. These included maps of soil parameters, topology, rainfall, settlements, roads, population density, etc. Information for each inland valley was extracted using the location information of the sites and the values were added to the dataset of questionnaire responses and observations.

Table 2 provides on overview of the 65 parameters in the dataset and their source (whether from the field surveys or public domain sources).

## Appendix B Supplementary materials

The following KMZ files contain the Google maps of the most important areas described in this article.

Map KMZ file containing the Google map.
