# Supplementary material for: A geospatial dataset of inland valleys in four zones in Benin, Sierra Leone and Mali
Source: Data Brief. 2019 Jan 22;23:103699. doi: 10.1016/j.dib.2019.103699 (PMC6383130; doi:10.1016/j.dib.2019.103699)
Supplement: Supplementary file 1 — Supplementary material [file mmc1.docx]

January 2, 2019 – Accra, Ghana

Concerns: Conflict of Interest and Authorship Conformation

The authors of the submission ‘*A geospatial dataset of inland valleys in four zones in Benin, Sierra Leone and Mali*’ (DIB-D-18-03247) to Data in Brief declare the following:

- All authors have participated in (a) conception and design, or analysis and interpretation of the data; (b) drafting the article or revising it critically for important intellectual content; and (c) approval of the final version.
- This manuscript has not been submitted to, nor is under review at, another journal or other publishing venue.
- The authors have no affiliation with any organization with a direct or indirect financial interest in the subject matter discussed in the manuscript

On behalf of all authors,


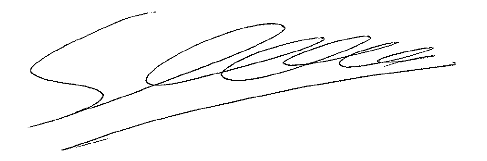


Sander Zwart

**Sander Zwart |** Coordinator TAAT Water Enabler Compact


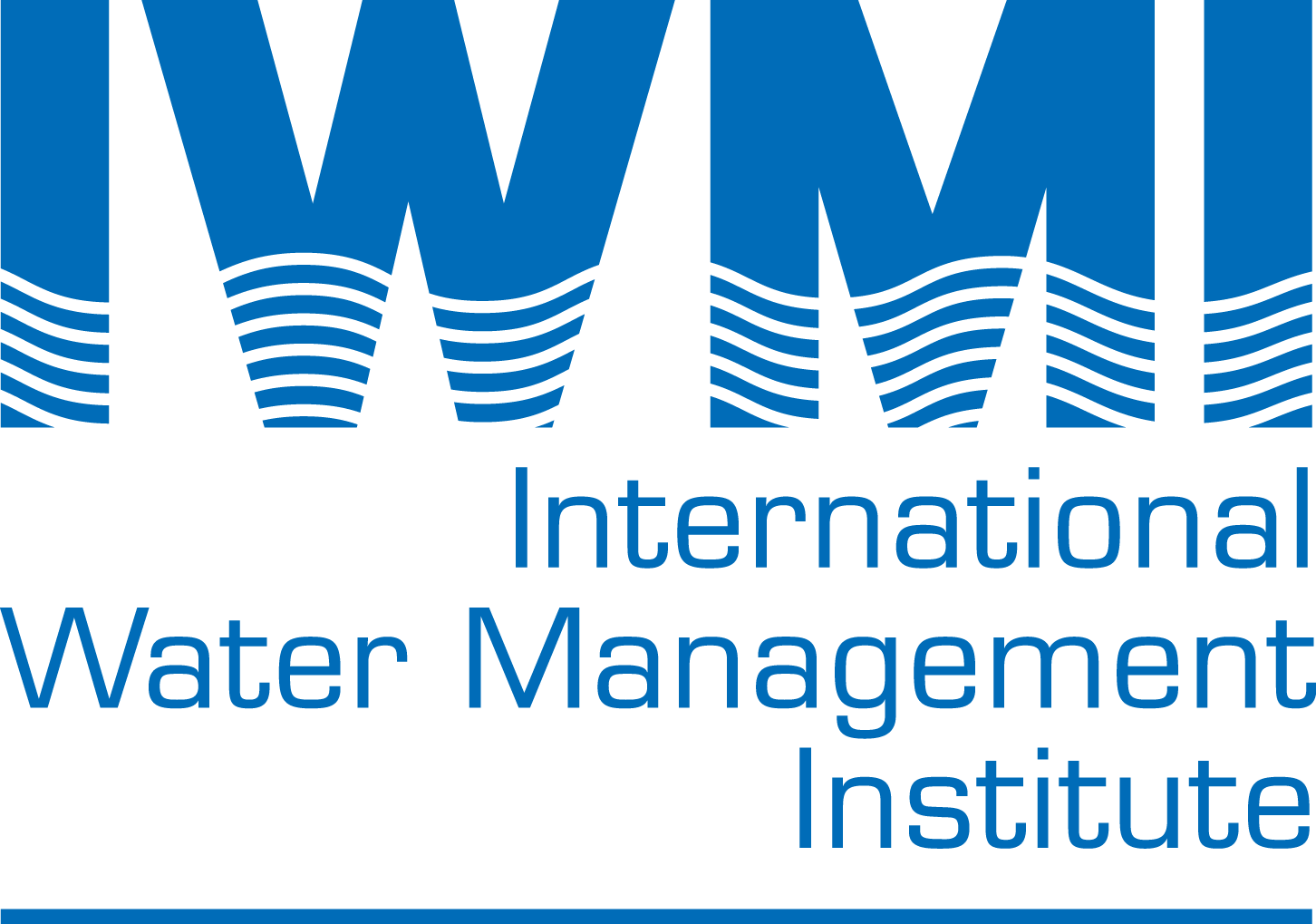

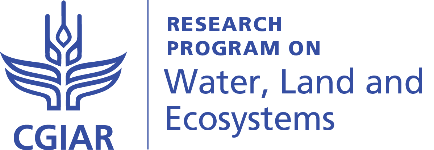

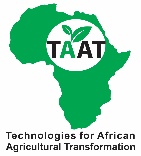


**International Water Management Institute**PMB CT 112 | Cantonments | Accra | Ghana
Tel +233 302 784 753 | Mobile +233 54 693 5497 | Skype rednasz
